# Attention-Guided Edge-Optimized Network for Real-Time Detection and Counting of Pre-Weaning Piglets in Farrowing Crates

**DOI:** 10.3390/ani15172553

**Published:** 2025-08-30

**Authors:** Ning Kong, Tongshuai Liu, Guoming Li, Lei Xi, Shuo Wang, Yuepeng Shi

**Affiliations:** 1 School of Energy and Intelligence Engineering, Henan University of Animal Husbandry and Economy, Zhengzhou 450046, China; 2 College of Animal Science & Technology, Henan University of Animal Husbandry and Economy, Zhengzhou 450046, China; 3 Henan Engineering Research Center on Animal Healthy Environment and Intelligent Equipment, Zhengzhou 450046, China; 4 Department of Poultry Science, The University of Georgia, Athens, GA 30602, USA; 5 Institute for Artificial Intelligence, The University of Georgia, Athens, GA 30602, USA; 6 Science and Technology Division, Henan University of Animal Husbandry and Economy, Zhengzhou 450046, China

**Keywords:** piglet counting, YOLO network, architecture optimization, lightweight deployment

## Abstract

Accurate, real-time, and cost-effective detection and counting of pre-weaning piglets are critical for improving piglet survival rates. However, achieving this remains technically challenging due to high computational demands, frequent occlusion, social behaviors, and cluttered backgrounds in commercial farming environments. To address these challenges, this study proposes a lightweight and attention-enhanced piglet detection and counting network based on an improved YOLOv8n architecture. The design includes three key innovations: (i) the standard C2f modules in the backbone were replaced with an efficient novel Multi-Scale Spatial Pyramid Attention (MSPA) module to enhance the multi-scale feature representation while a maintaining low computational cost; (ii) an improved Gather-and-Distribute (GD) mechanism was incorporated into the neck to facilitate feature fusion and accelerate inference; and (iii) the detection head and the sample assignment strategy were optimized to align the classification and localization tasks better, thereby improving the overall performance. Experiments on the custom dataset demonstrated the model’s superiority over state-of-the-art counterparts, achieving 88.5% precision and a 93.8% $mAP_{0.5}$. Furthermore, ablation studies showed that the model reduced the parameters, floating point operations (FLOPs), and model size by 58.45%, 46.91% and 56.45% compared to those of the baseline YOLOv8n, respectively, while achieving a 2.6% improvement in the detection precision and a 4.41% reduction in the counting MAE. The trained model was deployed on a Raspberry Pi 4B with ncnn to verify the effectiveness of the lightweight design, reaching an average inference speed of <87 ms per image. These findings confirm that the proposed method offers a practical, scalable solution for intelligent pig farming, combining a high accuracy, efficiency, and real-time performance in resource-limited environments.

## Simple Summary

To improve the survival and management of pre-weaning piglets, it is necessary to achieve accurate and real-time detection and counting in farrowing crates. However, frequent occlusion of the piglets, social behaviors, and cluttered backgrounds make this task difficult, especially when using lightweight models in resource-limited environments. In this study, we propose an improved piglet detection model based on YOLOv8n. The model replaces the original backbone module with a Multi-Scale Spatial Pyramid Attention (MSPA) module; introduces an improved Gather-and-Distribute (GD) mechanism in the neck; and optimizes the detection head and the sample assignment strategy. The experimental results show that compared with the baseline YOLOv8n, our model reduces the parameters, floating point operations, and model size by 58.45%, 46.91%, and 56.45%, respectively, while increasing the detection precision by 2.6% and reducing the counting error by 4.41%. In addition, the model was successfully deployed on a Raspberry Pi 4B, achieving an average inference speed of less than 87 ms per image. These results demonstrate that the proposed method achieves both high accuracy and a lightweight performance, providing a practical solution for intelligent pig farming.

## 1. Introduction

Accurate detection and counting of pre-weaning piglets are essential for improving a key performance indicator in swine production—the number of weaned piglets per sow per year, which reflects the overall breeding efficiency of the industry. On the one hand, piglet detection and counting can be utilized to measure the farrowing interval and duration of sows, determine the birth order of piglets, and calculate the litter size, thereby assessing the reproductive performance of sows and providing support for piglet weight measurements and behavior recognition [1,2,3]. On the other hand, through automated piglet detection and counting, combined with a behavioral analysis of the sows, it is possible to promptly detect events that affect the survival rate of pre-weaning piglets, such as crushing and suffocation [4,5,6,7], thereby enhancing the economic efficiency of the pig farming industry.

However, there are several significant challenges in solving these problems in real-world, practical environments. First, in farrowing crates, piglets live with the sow until weaning to facilitate nursing. Due to frequent movement, piglets are often occluded. Secondly, due to the social characteristics of mammals, pre-weaning piglets tend to gather and show group attachment. Finally, there are unstable environmental factors such as a varying light intensity caused by heating lamps. These factors (e.g., occlusion, group attachment, and the environment) collectively complicate the task of piglet detection and counting.

The traditional methods for this purpose rely on manual inspections. However, due to the unpredictability of sow farrowing times, the prolonged duration of the farrowing process, large litter sizes, and frequent movement of the piglets and severe occlusion, manual observation is time-consuming and labor-intensive. Additionally, the presence of humans in these facilities may bring pathogenic bacteria [8], posing health risks to the animals. The widespread nature of livestock monitoring systems and advancements in computational technologies have provided new avenues for addressing these challenges.

Piglet detection and counting can be formulated as a computer vision task and addressed through artificial-intelligence-based detection methods. The current research in this field can be categorized according to three main directions: image analysis, machine vision, and deep learning. Image analysis methods for solving such problems typically involve following two steps: constructing specific algorithms and obtaining the results using software like MATLAB R2019b. Lu et al. [1] proposed a segmentation algorithm for gray-scale images of gathered piglets based on ellipse fitting. By fitting ellipses to the contours of individual and clustered piglets, rules for merging these elliptical contours were extracted. After applying these rules to the images, the number of ellipses corresponds to the number of piglets. The results showed an accuracy of over 86% when the number of piglets was less than seven in one gray-scale image. Similarly, Oczak et al. [2] recorded and analyzed a video of the sow farrowing process, marked timestamps when piglets were born, and then converted the video stream into images. Image analysis technology was used to segment objects and extract three parameters: the number of piglets, the number of pixels contained in the piglet covering area, and the perimeter of the piglet’s boundary. Finally, a Transfer Function model was established based on these three parameters to estimate the number of piglets in the farrowing crates, achieving a standard deviation of 1.73 on a test set of 34 piglets. Nevertheless, the application of image analysis technology to piglet detection and counting remains constrained by its offline nature, rendering it incapable of meeting real-time processing requirements.

Machine vision methods emphasize the integration of hardware and image processing algorithms. Pastell et al. [9] used wireless 3D accelerometers on 29 crated and 33 pen-housed sows, analyzing the activity trends via a dynamic linear model and detecting pre-farrowing spikes with a CUSUM chart. The system identified activity increases of 13±4.8 h before farrowing (96.7% sensitivity, 100% specificity), demonstrating feasibility across housing systems. Zhang et al. [10] utilized a self-developed infrared image acquisition device to capture videos of sow farrowing. After edge detection, binarization, filtering, denoising, and segmentation of the video images, the recognition accuracy for newborn piglets reached 95.5%. Silapachote et al. [11] established a sow farrowing monitoring system using a Raspberry Pi and a camera. By applying three image processing steps, including equalization of histograms to the video frames, an automatic detection system for piglet births was established. Despite these advances, the high computational demands and low cost effectiveness of machine-vision-based systems hinder their widespread adoption.

In recent years, with the development of deep learning, many machine vision tasks have gradually shifted from traditional algorithms to deep learning models such as Mask R-CNN and YOLO [12,13,14]. Tian et al. [15] proposed a deep learning network that was more suitable for counting pigs by integrating a Counting CNN and ResNeXt. On a real dataset consisting of 485 images with an average of 15 pigs per image, the results showed an average absolute error of 1.67 in pig counting and an inference time of 42 ms per frame. Huang et al. [16] developed a two-stage Center Clustering Network (CClusNet) for automated piglet counting under occlusion, implementing a systematic framework comprising three computational phases. Initially, a semantic segmentation map and a center offset vector map are generated for each image. Then, the discrete center points in the two maps are generated through clustering. Finally, the center points are input to the mean shift algorithm to acquire the result. Their demonstration showed that the CClusnet had an average absolute error of 0.43 in piglet counting tasks with an inference speed of 4.3 s per frame. Zhang et al. [17] introduced an efficient global attention module into the YOLOv8 model and built a pig counting system named YOLOv8x-Ours. Then, the model was embedded into a WeChat applet for testing. The average absolute error on the test set was 1.72. He et al. [18] employed an SPD-Conv structure in the backbone of YOLOv7, replaced the neck part with an AFPN, used a rotating bounding box on the head, and built a PDC-YOLO network to count pigs in complex environments. On the custom dataset, the average counting accuracy reached 91.97%. Zhou et al. [19] first used a track inspection robot located above the farrowing pen to collect and store images and then detected the posture of the sows based on the YOLOv8 model. The accuracy rate and the processing speed reached 97.08% and 36.4 FPS, respectively. Meanwhile, based on a Temporal Shift Module (TSM), the dynamic behavior of the piglets was recognized with a highest accuracy of 93.61%. The fastest inference speed for a single video clip on the Jetson Nano was reduced to 542 ms.

While deep learning has become a mainstream solution for this task, certain limitations remain in the existing approaches. First, a large number of studies have focused on individual counting, behavioral recognition, farrowing detection, and early warnings about weak pigs [20,21,22], while there have been few studies on the individual detection and counting of pre-weaning piglets. The detection and counting results in the existing research still have room for improvement. Additionally, some detection models based on deep learning are two-stage or need depth information for assistance, resulting in high computational costs that hinder their ability to meet real-time detection needs [23,24]. Furthermore, few studies have identified the differences between newborn piglets and pre-weaned piglets, despite the fact that such differentiation is important for improving the survival rate of newborn piglets and enhancing the welfare of sows.

**Figure 1:**
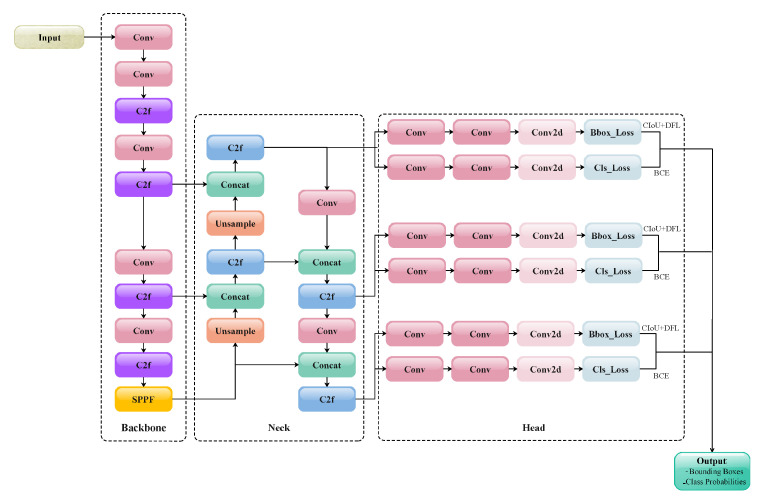
Overall structure of YOLOv8 model.

As a representative object detection framework in deep learning, YOLOv8 has been widely adopted and established as a mainstream baseline model owing to its well-balanced performance in terms of its real-time speed, accuracy, and flexibility. Although newer versions such as YOLOv12 have been released, YOLOv8 continues to demonstrate excellent stability and scalability [25,26]. The structure of the YOLOv8 model is shown in Figure 1, which can be divided into three parts: the backbone, neck, and head. The composition of each part of the network and the design of the order of modules are driven by theory and a large number of experiments aiming to balance the performance and computational efficiency. The backbone part is responsible for feature extraction, while the neck part further fuses the extracted features and integrates multi-scale features. Finally, the head part performs bounding box regression and category prediction for different-scale feature maps in parallel.

As the nano version of the YOLOv8 series, YOLOv8n has demonstrated superior computational efficiency and reduced parameterization while maintaining a competitive detection accuracy, rendering it particularly advantageous for edge-computing deployments with constrained computational resources; however, this performance optimization entails a fundamental trade-off in cross-domain adaptability, manifesting as a visible performance degradation when applied to non-canonical datasets [27,28,29].

**Figure 2:**
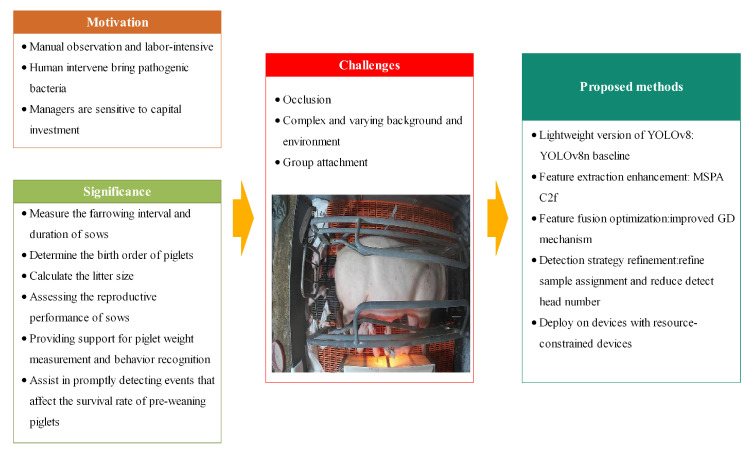
A research map of the proposed piglet detection and counting model.

This paper aims to meet the needs of real-time piglet detection and counting by improving the cost effectiveness of the system and ensuring the accuracy and inference speed. Specifically, a single-stage lightweight algorithm named MGDT-YOLO is proposed, which is based on the YOLOv8n model. By redesigning and employing more efficient modules, MGDT-YOLO has achieved relatively ideal results in terms of the precision, parameters, FLOPs, and model size. A research map of the proposed model is shown in Figure 2.

The main contributions of this paper are summarized as follows:

- Feature extraction enhancement: A concise and effective multi-scale spatial pyramid attention C2f module is proposed. By replacing the C2f module in the backbone, the ability of the YOLOv8n backbone to extract multi-scale spatial information on the input is improved, enabling it to fully integrate structural regularization and structural information and efficiently establish long-distance channel dependencies.
- Neck structure optimization: An improved Gather-and-Distribute mechanism is incorporated into the neck part of YOLOv8n, which enables and accelerates multi-scale feature fusion by fully leveraging high-level semantic features and low-level spatial information, thereby improving the detection speed of the model.
- Detection strategy refinement: The number of detection heads is reduced to one, and both the sample assignment strategy and the detection head structure are refined, effectively reducing the number of parameters while maintaining or even improving the detection performance.

## 2. Materials

The dataset used in this paper comprises two components. The first part consists of video recordings capturing the daily behavior of periparturient and lactating sows collected between 17 November 2023 and 30 January 2024 in the experimental farrowing crates of a commercial pig farm in Qi County, Hebi City, Henan Province. The recordings were obtained using an EZVIZ CS-C5S-3C2WFR camera mounted above the aisle behind the farrowing pens, with a resolution of 1920×1080 pixels, which covered typical challenges such as piglet occlusion, clustering, movement, and overexposure caused by heat lamps. The video frames were extracted at 10 s intervals, and a subsequent manual selection process yielded 221 valid images. These images were then annotated using the Segment Anything Model (SAM) [30]. Compared with traditional labeling tools such as Labelme and LabelImg, the SAM offered higher efficiency through semi-automated annotation and improved accuracy.

The second part of the dataset was obtained from the open-access platform Roboflow [31]. The statistical analysis indicated that most of the images were captured on several specific days in June 2024, November 2024, and February 2025. This dataset contained piglet images from a top-down perspective of farrowing pens, each with a resolution of 1920×1080 pixels. The downloaded images were manually reviewed and further filtered, resulting in the selection of 2000 representative images.

Following dataset integration, our study utilized a final collection of 2221 images, which were randomly partitioned into training (75%), validation (15%), and test (10%) sets using an independent script. This split was carried out only once and was kept unchanged throughout all experiments. Therefore, all models were trained and evaluated on the exact same dataset partitions, ensuring reproducibility under identical conditions. The test set consisted of 220 images containing 1320 annotated piglets, averaging 6 piglets per image. Conventional approaches have often applied offline data augmentation before training to enhance the model’s robustness and generalization. However, this process produced redundant image files, consuming extra storage and potentially causing labeling errors during manual annotation synchronization. This paper adopted a more elegant method leveraging Ultralytics (v8.0.120), which incorporated the albumentations library for online data augmentation during training. This framework enabled seamless customization of the augmentation parameters—including flipping, crop, brightness adjustment, mosaic transformations, etc.—via configuration files, thus improving both the training flexibility and efficiency. Next, the images were annotated into two categories: piglets capable of normal movement (class_0) and piglets in the process of being born from the sow’s birth canal (class_1). This categorization not only fulfilled the intended task but also facilitated the identification of sow farrowing events, thereby offering technical support for reducing piglet mortality and optimizing postpartum sow management. Importantly, each image naturally contains both class_0 and class_1 piglets, reflecting real-world scenarios. Therefore, each subset preserves the relative proportions of both classes without requiring explicit stratified splitting, ensuring a representative and reliable evaluation of the model’s performance.

**Figure 3:**
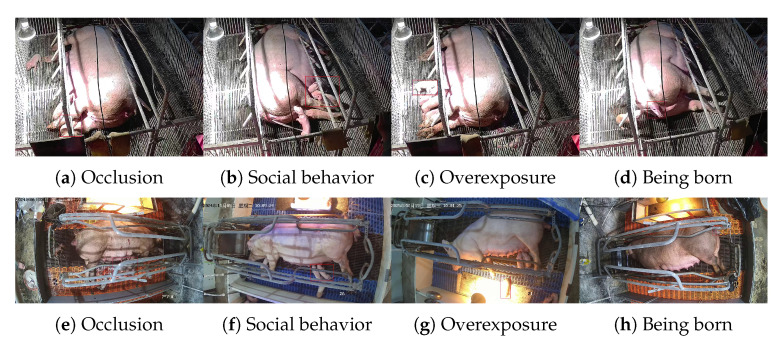
Samples of piglets under different conditions. The top row shows examples from the self-recorded dataset (rear-view angle). The bottom row presents examples from the open-source dataset (top-down perspective).

The dataset encompasses various challenging scenarios, including occlusion, clustering, movement, overexposure caused by heat lamps, and different viewing angles, as illustrated in Figure 3. A statistical analysis revealed that 2037 of the total 2221 images contained occlusion, indicating that in over 91% of the images, at least one piglet was partially blocked by the farrowing crate. In addition, 252 images exhibited overexposure, primarily occurring during low-temperature periods between 9:00 and 11:00 a.m. These proportions reflect the typical challenges of real-world farming environments and ensure the robustness of the model evaluation. The annotation results indicated that the dataset contained 11,162 samples of class_0 and 2096 samples of class_1, resulting in an expected class imbalance. This imbalance was not mitigated because the class_0 piglets exhibited significantly more distinctive features compared those in to class_1, allowing the model to learn effectively despite the disparity in the sample numbers.

## 3. Methodology

**Figure 4:**
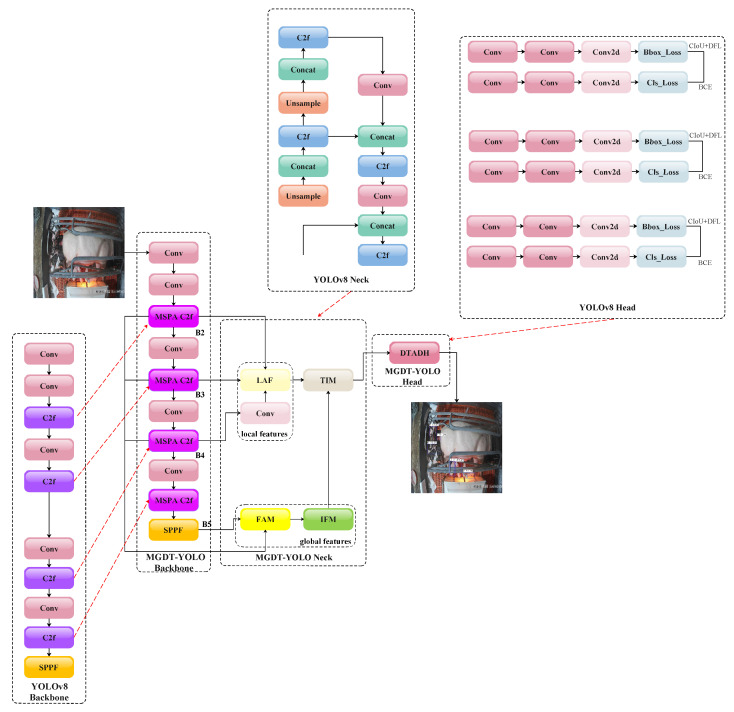
The overall network framework of MGDT-YOLO. Compared with the original YOLOv8n pipeline (see Figure 1), MGDT-YOLO introduces three key improvements: (i) the standard C2f module in the backbone is replaced with the proposed MSPA module for enhanced multi-scale feature representation; (ii) the neck is optimized with a Gather-and-Distribute (GD) mechanism to improve the feature fusion; and (iii) the detection head is simplified into a task-aligned design for better classification–localization consistency. Red arrows in the figure indicate the corresponding modifications relative to YOLOv8.

This section elaborates on the architecture of MGDT-YOLO, which comprises three key components: an enhanced backbone, an optimized neck, and a refined task-aligned detection head. The first section presents the MSPA C2f module in the backbone, which enhances the multi-scale feature representation through hierarchical spatial–channel interactions. Then, the second part introduces a refined Gather-and-Distribute mechanism in the neck architecture, establishing bidirectional cross-scale connections to significantly improve the feature fusion efficiency across different semantic levels. The final section details the detection head’s refined strategy that aligns classification and regression tasks through structural optimization and heuristically selecting high-quality anchors. The overall framework of our model is shown in Figure 4.

### 3.1. Multi-Scale Spatial Pyramid Attention C2f

**Figure 5:**
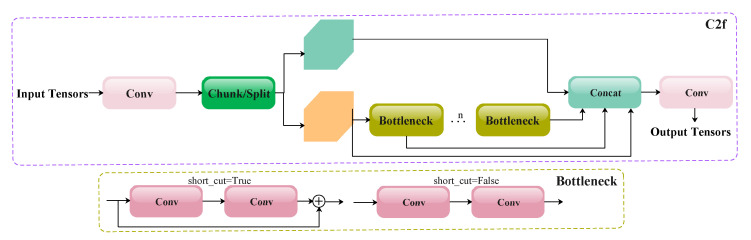
The structure of the C2f module in the YOLOv8 network. Light-colored Conv blocks in this paper represent convolutional layers with 1×1 kernels, and dark-colored Conv blocks correspond to layers employing 3×3 kernels.

An analysis of the network structure and the official documentation reveal that C2f is a key module in the backbone of YOLOv8. C2f, an abbreviation for a faster implementation of the CSP bottleneck with two convolutions, typically appears in pairs with the Conv module. It enhances the feature extraction capabilities by capturing complex patterns in the input data, which is crucial for improving the model’s detection performance. The structure of the C2f module is illustrated in Figure 5.

C2f integrates 1×1 and 3×3 convolutions to enhance the global receptive field through cross-layer information fusion while preserving the local receptive field. Specifically, the 1×1 convolution first reduces the number of channels in the feature maps, followed by 3×3 convolution to refine the feature representation. This design effectively decreases the number of parameters and the computational overhead, ensuring high computational efficiency. However, the introduction of 1×1 convolution may lead to the loss of fine-grained feature information, while the 3×3 convolution in the CSP bottleneck has inherent limitations in expanding the receptive field for specific target processing. Additionally, both the structural diagram and the source code implementation of C2f indicate constraints in multi-scale feature representation and channel interaction.

**Figure 6:**
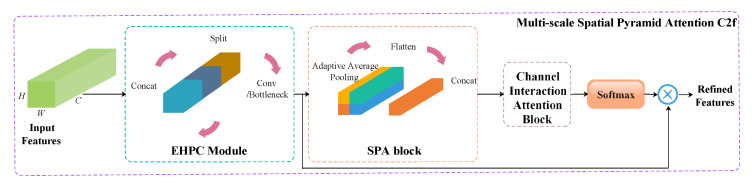
The architecture of the proposed MSPA C2f.

To address the challenges posed by complex backgrounds and environments in real-world farrowing pens for piglet detection, enhancing the model’s feature extraction capability is a viable and effective solution. Based on the aforementioned analysis and drawing inspiration from the work of [32], this paper proposes a novel C2f module enhanced with multi-scale spatial pyramid attention. The structure of MSPA C2f, as illustrated in Figure 6, consists of four core functional modules: the Enhanced Hierarchical-Phantom Convolution module (EHPC), the Spatial Pyramid Aggregation (SPA) block, the Channel Interaction Attention (CIA) block, and the Softmax function.

#### 3.1.1. The Enhanced Hierarchical-Phantom Convolution Module

**Figure 7:**
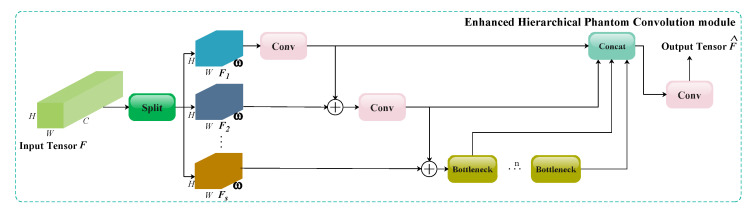
The data flowchart of the EHPC module.

The structure of the EHPC module is illustrated in Figure 7 and consists of four sequential operations: Split, Conv/Bottleneck, element-wise summation, and concatenation. Unlike the traditional C2f module, which first applies a 1×1 convolution to the input before performing multi-scale feature extraction layer by layer, the EHPC module initially partitions the input into multiple chunks and subsequently performs feature extraction and aggregation accordingly.

Specifically, let the input of MSPA C2f be represented as $F\in R^{C\times H\times W}$. F is evenly partitioned along the channel dimension into three subcomponents, denoted as $F_{i}\in R^{\omega \times H\times W}$, where i∈{1,2,3}. The number of chunks is set to 3 based on prior studies [32] which systematically evaluated values ranging from 2 to 5 and found that the performance did not increase monotonically but reached its optimal value at 3. In addition, since the original C2f modules in YOLOv8 retain an unchanged feature scale, choosing 3 chunks allows MSPA C2f to be seamlessly integrated into the backbone without disrupting the overall feature flow. Subsequently, the following operations are performed:

(1) $$\begin{array}{cc} \; & \hat{F_{1}}=Conv_{1}\left(F_{1}\right)\\ \; & \hat{F_{2}}=Conv_{2}\left(\hat{F_{1}}⊕F_{2}\right)\\ \; & \hat{F_{3}}=Bottleneck\left(\hat{F_{2}}⊕F_{3}\right) \end{array}$$

where $\hat{F_{i}}\in R^{\omega \times H\times W}$, i∈1,2,3 represents the enhanced output feature subsets. Conv_1 and Conv_2 correspond to 1×1 convolutional layers, which not only extract local feature information but also perform channel compression on the input to reduce the computational complexity. The bottleneck module, which is inherited from the original C2f structure, applies consecutive 3×3 convolution layers along with residual connections to expand the receptive field of the output feature maps. The operator ⊕ denotes pixel-wise summation, which enhances the multi-scale feature fusion by efficiently facilitating the propagation of information across layers. The output of the EHPC module is given by

(2) $$\hat{F}=Conv_{3}(Concat(\left[\hat{F_{1}},\hat{F_{2}},\hat{F_{3}}\right]))$$

where $\hat{F}\in R^{C\times H\times W}$. The concat operation aggregates $\hat{F_{1}},$$\hat{F_{2}}$, and $\hat{F_{3}}$ along the channel dimension. The 1×1 convolutional layer (Conv_3) ensures dimensional consistency in output channels, preserving structural compatibility with the baseline C2f.

In summary, the EHPC module employs a hierarchical multi-scale architecture to process input feature maps, jointly optimizing the local feature discrimination and global contextual awareness for improved feature representation.

#### 3.1.2. Channel Relationship Modeling

The work [33] demonstrated that modeling the interdependencies among feature channels can significantly enhance the performance of lightweight networks. While the capability of the EHPC module to model channel relationships is limited, the SPA block and the CIA block are introduced to perform spatial feature aggregation and channel weight allocation, respectively. This integration enables comprehensive modeling of the feature channel relationships, thereby enhancing the overall feature representation capability of the model.

**Figure 8:**
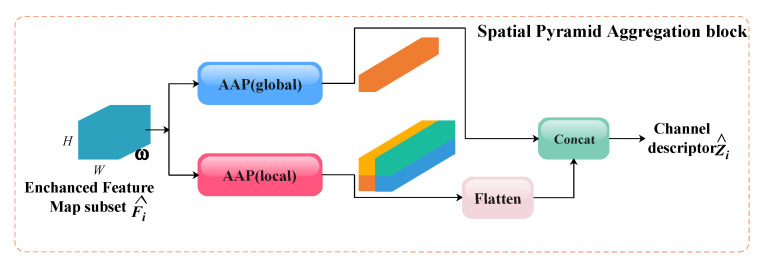
A schematic diagram of the SPA block.

The structure of the SPA block is illustrated in Figure 8. Continuing with the notation in the previous section, the input to this block is the enhanced output feature subset $\hat{F_{i}}$ from the EHPC module, and the output is defined as follows:

(3) $$\hat{Z_{i}}=SPA\left({\hat{F}}_{i}\right)=Concat(AAP_{global}\left({\hat{F}}_{i},1\right),Flatten\left(AAP_{local}\left({\hat{F}}_{i},2\right)\right))$$

where $AAP_{global}$ and $AAP_{local}$ denote adaptive average pooling with kernel sizes of 1×1 and 2×2, respectively, which are employed to extract global and local feature information. Subsequently, the local features $\left(AAP_{local}\left({\hat{F}}_{i},2\right)\right)$ are flattened along the channel dimension. Finally, the global and local information is concatenated along the channel dimension.

**Figure 9:**
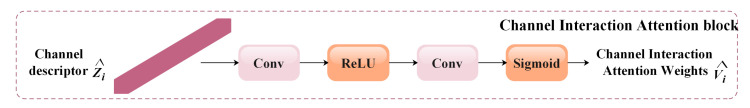
The architecture of a CIA block.

The CIA block forms the core mechanism for channel relationship modeling, performing two critical functions: (1) adjusting the dimension of the feature output from the SPA block and (2) generating channel-wise weights. The structure of the CIA block is illustrated in Figure 9. Given an input $Z_{i}$, the output is formulated as follows:

(4) $$\hat{V_{i}}=\sigma (Conv_{4}(ReLU(Conv_{5}(\hat{Z_{i}}))))$$

where $\hat{V_{i}}\in R^{\omega \times H\times W}$, and Conv_4 and Conv_5 represent the 1×1 convolution layers for up-sampling and down-sampling, respectively. The activation functions σ and ReLU correspond to the Sigmoid and Re-LU functions. The output $\hat{V_{i}}$ of the CIA block is subsequently processed through a Softmax layer to generate the final feature channel weights. This design enables precise channel-wise feature recalibration, effectively enhancing the model’s capacity to capture the channel dependencies and optimize feature utilization.

To demonstrate the effectiveness of the design, we first compared the parameters and the computational complexity of MSPA C2f and C2f. The results are shown in Table 1. Further experimental details are presented in Section 4.2.1.

### 3.2. Redesign of the Neck Applying the Gather-and-Distribute Mechanism

The neck of YOLOv8 employs a combination of a Feature Pyramid Network (FPN) and a Path Aggregation Network (PAN) to achieve multi-scale feature fusion. This architectural design significantly enhances the expressive capability of the features, thereby improving the robustness of object detection. However, the integration of the FPN and PAN also introduces limitations, including increased computational complexity and the potential loss of information during transmission. Specifically, feature information is constrained to propagate only between adjacent layers, and cross-layer fusion necessitates selection and transfer through intermediate layers. As a result, the overall effectiveness of the information fusion within the neck is somewhat compromised.

Gold-YOLO, introduced by Huawei Noah’s Ark Lab in 2023 [34], reengineered the neck part of the original network and innovatively proposed the Gather-and-Distribute (GD) mechanism to enhance the effectiveness and efficiency of multi-scale feature fusion while minimizing unnecessary information loss. The GD mechanism comprises four core modules: the Feature Alignment Module (FAM), the Information Fusion Module (IFM), the Lightweight Adjacent Layer Fusion Module (LAF), and the Information Injection Module (Inject). Specifically, multi-scale features are aggregated through the FAM, IFM, and LAF, which eliminate redundant information and significantly reduce the computational resource consumption. The Inject module enhances the feature representation further by integrating global and local feature information, enabling efficient feature distribution and enhancement. This design is particularly well suited to target detection tasks that require strong contextual dependencies, offering improved performance and computational efficiency.

**Figure 10:**
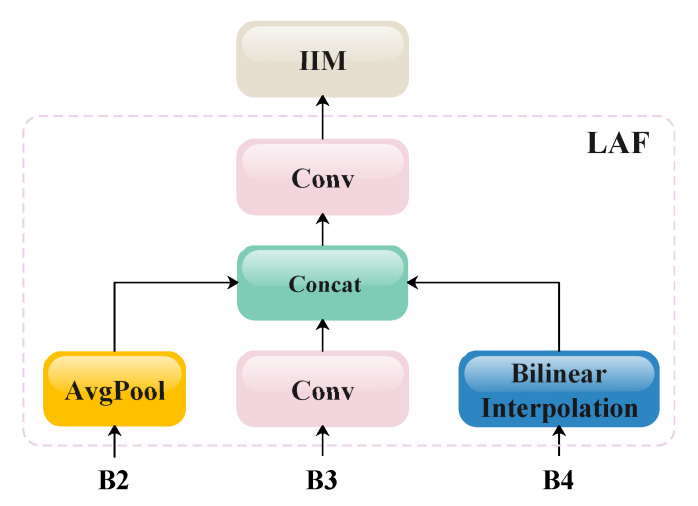
A schematic diagram of the LAF. AvgPool means average pooling, and Bilinear Interpolation indicates the bilinear interpolation algorithm. Concat stands for the concatenation operation along the channel dimension. AvgPool, Conv, and Bilinear Interpolation unify the sizes of B2, B3, and B4.

**Figure 11:**
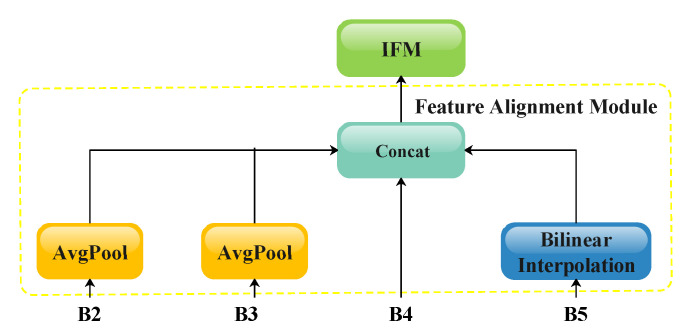
A schematic illustration of the FAM. The dimensions of B2, B3, B4, and B5 are aligned using AvgPool and bilinear interpolation.

**Figure 12:**
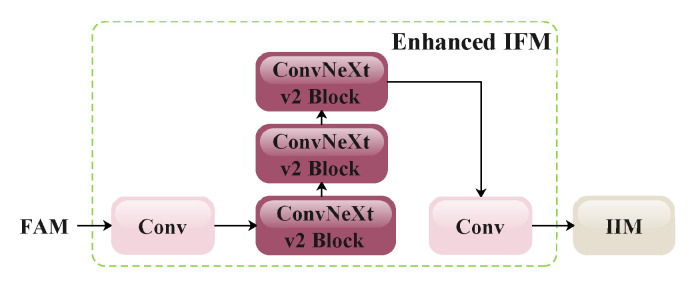
A schematic illustration of the improved IFM.

**Figure 13:**
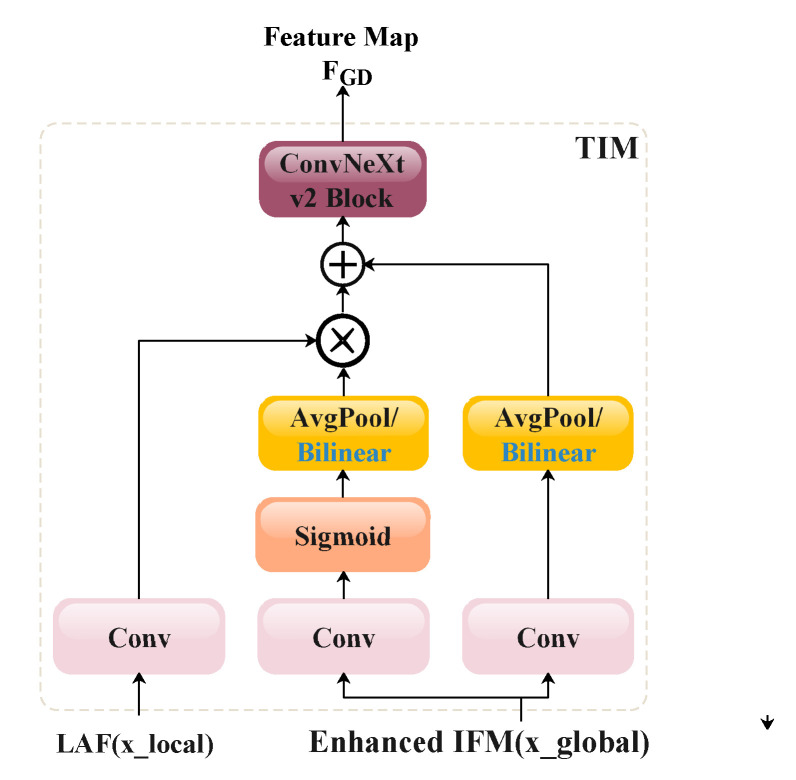
A schematic illustration of the proposed TIM. AvgPool or bilinear interpolation is selected based on the size relationship of the global and local feature.

Considering that the target size in piglet detection and counting scenarios exhibits minimal variation, this study adopts the design principles of the GD-YOLOv8 [35] neck to balance the detection accuracy and computational overhead. Figure 4 shows an overview of our optimized neck architecture, while Figure 10, Figure 11, Figure 12 and Figure 13 provide detailed schematics of each constituent module. Let the feature tensors extracted from the backbone after each Conv/C2f pair processing be denoted as B2, B3, B4, and B5. Initially, the features B2, B3, and B4 from shallower levels are fed into the LAF module to generate local features. Subsequently, the features B2, B3, B4, and B5 from all four scales are processed through the FAM for size alignment and then input into the IFM to form the global features. Both the local and global features are then directed to the Integrated Information Injection Module (TIM), which outputs the final feature map $F_{GD}$.

**Figure 14:**
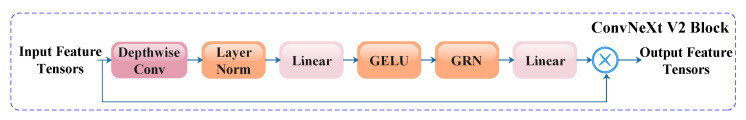
The structure of the ConvNeXt V2 block.

This paper differs from GD-YOLOv8; where the RepVGG block in the original IFM offers a fast inference speed, it lacks the capability to model the feature context, especially in capturing long-range dependencies among features. To address this limitation, this paper introduces the ConvNeXt V2 block into the original IFM and yields the improved IFM. ConvNeXt V2, proposed by [36] from Facebook in 2023, is an advanced convolutional module built upon ResNet and incorporates principles from transformer architectures. The ConvNeXt V2 block is the core component of the ConvNeXt V2 network, and the structure is illustrated in Figure 14. By adopting a transformer-like design, the block demonstrates significant performance improvements over those of the conventional RepVGG block. Equally, its exclusive use for standard convolutional operations ensures ease of integration and scalability. The incorporation of the normalization layer and Gaussian error linear unit activation functions enhances the training stability and convergence speed further.

The ConvNeXt V2 block is also applied at the end of the TIM to refine the feature representation. Additionally, the primary difference between the TIM and the Inject module in GD-YOLOv8 is that the global features are not split but instead copied directly to two branches. In one branch, the global features go through the Sigmoid layer and interact with the local features to form an attention mechanism, while the other branch adopts a residual-like connection. A 1×1 convolution operation is employed to fuse the information across feature channels, and size alignment between local and global features is achieved using either average pooling (AvgPool) or bilinear interpolation (Bilinear). When the global feature tensors are larger than the local features, pooling operations are used for size alignment; otherwise, interpolation is applied to ensure proper pixel-wise summation in the next step.

### 3.3. The Task-Aligned Detection Head

Generally, the detection head of YOLOv8 is designed in a decoupled manner. As shown in Figure 1, the inputs to the classification and regression branches are the multi-scale feature maps generated by the neck. Subsequently, the two branches perform category prediction, bounding box regression, and target confidence prediction, respectively. This design allows each branch to focus more effectively on its specific task, thereby enhancing the accuracy and stability of detection. However, as highlighted in [37,38], the decoupled detection head may introduce two significant issues. First, inconsistencies may arise between the results of the classification and regression tasks. Second, task-agnostic sample assignment, which is often caused by factors such as irregular target shapes, may lead to scenarios where an accurately predicted regression box is suppressed by another less accurate bounding box during the Non-Maximum Suppression process.

**Figure 15:**
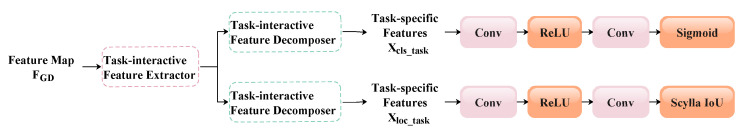
The schematic structure of the proposed THead.

**Figure 16:**
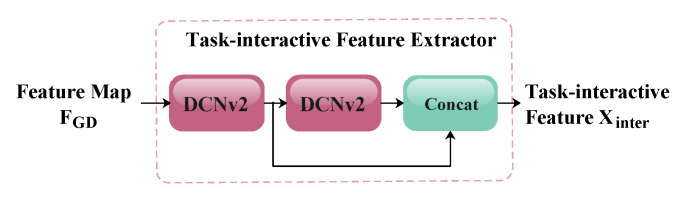
An illustration of Task-Interactive Feature Extractor.

**Figure 17:**
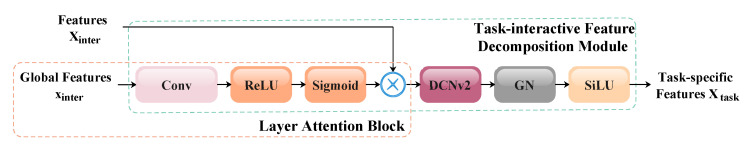
An illustration of the Task-Interactive Feature Decomposition Module.

To address the aforementioned issues, Feng et al. [37] proposed a task-aligned head and a task alignment learning algorithm. By designing a task-aligned detection head structure, optimizing the task assignment for training samples, and introducing a novel task alignment loss function, this study achieved a superior performance in one-stage object detection. Inspired by these design principles, this study reconstructs the YOLOv8n detection head with lightweight optimizations to improve the computational efficiency without compromising accuracy. As illustrated in Figure 4 and Figure 15, MGDT-YOLO first reduces the number of detection heads to one, followed by simplification of the shared feature extractor structure, which is shown in Figure 16. This simplification reduces the number of original convolutional modules and replaces the standard convolution with Deformable Convolutional Networks V2 (DCN V2), which is more suitable for task alignment. The feature decomposition module output, as proposed in the Layer Attention Block by [37], is now enhanced through DCN V2 operations, as illustrated in Figure 17. The use of DCNv2 in the detection head was motivated by its proven effectiveness in improving the task alignment between classification and localization, which is critical in occlusion object detection. Unlike conventional attention mechanisms (e.g., SE, CBAM), which mainly reweight the channel or spatial responses, DCNv2 introduces learnable offsets that allow for more flexible geometric modeling of objects with varying poses and shapes. This property enables the detection head to adapt to the inherent variability in piglets’ appearance and the occlusion conditions better. Prior studies such as OD-YOLO [39] have demonstrated that integrating DCNv2 into the feature decomposition module yields a superior performance compared to that of alternative attention strategies. Therefore, DCNv2 was chosen as it provides a stronger balance between feature adaptivity and accurate localization, leading to an improved overall detection performance.

Moreover, this study optimizes the sample assignment strategy of the detection head. The existing method in YOLOv8 follows a dynamic adjustment process, wherein the alignment metric *t* of each anchor is computed cyclically. Subsequently, the top *k* anchors with the highest scores are selected as positive samples. The formula for *t* is

(5) $$t=s^{\alpha }\times u^{\beta }$$

where *s* is the classification score of the candidate bounding box, and *u* is the CIoU between the candidate box and the target box. α and β are constant hyperparameters that control the proportion of the classification performance and the regression performance in the alignment metric. This dynamic task alignment sample assignment is critical for the joint optimization of classification and regression tasks. However, this strategy presents a minor issue: during the early stages of model training, the classification confidence score *s* is not highly reliable. As training progresses, the classification results gradually become more stable and accurate. Consequently, in the initial training phase, the alignment metric *t* should be predominantly influenced by *u* to ensure the selection of reliable initial samples. As training goes, the contribution of *s* should progressively increase to facilitate high-quality sample allocation.

Based on this insight, this study proposes a heuristic dynamic adjustment algorithm for α, which is integrated with YOLOv8’s original task-aligned sample allocation strategy:

(6) α=default_alpha∗(max_epochs−current_epoch)/max_epochs.

where default_alpha uses the original fixed value of 0.5, s∈[0,1], current_epoch represents the current training iteration, and max_epochs denotes the total number of training epochs. The experimental results demonstrating the effectiveness of this approach are presented in Section 4.2 and Section 4.2.1.

## 4. Experiments and Result Analysis

This section first presents the experimental setup, followed by a comparative benchmark analysis against state-of-the-art approaches. Subsequently, comprehensive ablation studies are conducted to validate the contribution of the proposed components. Finally, the optimized model is deployed on computational-resource-constrained device to assess its real-world performance.

### 4.1. The Experimental Setting

The experimental environment of this study consists of both software and hardware components. The software environment includes Ubuntu 18.04 as the operating system, Python 3.9.0 as the programming language, and PyTorch 1.12.1 as the deep learning framework, with CUDA 11.3 and CuDNN 8.3.2 for GPU acceleration. The hardware platform comprises a 16-core Intel Core i9-11900K CPU and an NVIDIA GeForce RTX 3060Ti GPU with 8 GB of video memory. YOLOv8n, implemented using Ultralytics version 8.0.120, serves as the baseline model for this study. The key hyperparameters used during the training, including the number of training epochs, batch size, learning rate, and IoU threshold, are summarized in Table 2.

### 4.2. Quantitative Results

To comprehensively and accurately assess the rationality of the proposed model, this paper employs standard MS COCO evaluation metrics, including precision, recall, $mAP_{0.5}$, and mAP, as well as model lightweight metrics, namely the parameters, model size, FLOPs, and inference speed, for comparison with other SOTA models. The open-source MMDetection [40] framework from OpenMMLab is utilized for the rapid deployment, training, and performance evaluation of five benchmark models: Faster R-CNN [41], SSD_Lite [42], TOOD [37], ATSS DyHead [38], and YOLOX-tiny [43]. Training and evaluation of Gold YOLO [34] and the proposed model are conducted based on their official open-source implementations. Notably, due to the difficulty in fully aligning all of the hyperparameters across all comparison models, we conduct a separate evaluation of the model’s inference time. Moreover, the highest and lowest inference times for a single image are excluded when computing the average inference time on the test set.

The comparison results in Table 3 demonstrate that the proposed model exhibits a superior performance in piglet detection. In particular, compared to the representative two-stage network Faster R-CNN, MGDT-YOLO achieves a significant advantage in both its detection accuracy and model efficiency, where the detection accuracy is improved by 2.25 times, $mAP_{0.5}$ increases by 19.3%, and mAP is bolstered by 21.8%, while the model size and FLOP counts are merely 0.6% and 2.3% of Faster R-CNN’s respective values. Compared with other one-stage detection networks, MGDT-YOLO has a slightly longer inference time than that of only Gold YOLO while achieving significant improvements in the detection performance with a minimal computational overhead. At an IoU threshold of 0.5, MGDT-YOLO achieves the highest precision of 88.50% and the second highest $mAP_{0.5}$ at 93.80%, slightly lower than the latest Ultralytics YOLO11n [44] by only 0.4%. To validate the proposed lightweight YOLOv8 improvements further, we also included a comparison with RT-DETRv2 [45], a transformer-based detection model that has recently attracted significant attention, and conducted experiments on the Autodl platform equipped with an NVIDIA GeForce RTX 3090 (24 GB) GPU. Although RT-DETRv2 attains higher mAP (75.30%) and $mAP_{0.5}$ (94.80%) values, its computational cost is substantially higher, with 40.44 M parameters, 132.7 G FLOPs, and an inference time of 15.2 ms per image, making it less suitable for deployment on resource-constrained devices. In contrast, MGDT-YOLO achieves a competitive detection performance while maintaining a lightweight model (1.249 M parameters, 4.3 G FLOPs) and fast inference (8.0 ms), demonstrating an effective balance between accuracy and efficiency.

It is worth noting that although the proposed model has fewer parameters, lower FLOPs, and a smaller size compared to these values for Gold YOLO, its inference time is slightly longer. This is primarily due to the introduction of the attention mechanism into MSPA C2f and the replacement of standard convolutions with DCN V2 in THead, which enhance the model performance while also introducing additional matrix computations and pooling operations, resulting in an increased computational overhead.

#### 4.2.1. Ablation Studies

To evaluate the effectiveness of the proposed improvement, ablation studies were conducted to independently assess the contribution of each method. The detection performance was evaluated using precision, recall, $mAP_{0.5}$, mAP, parameters, model size, FLOPs, and inference speed, with the results presented in Table 4. The counting performance was evaluated using three key metrics: the mean absolute error (MAE), mean square error (MSE), and mean accuracy rate (MAR). The definitions of the MAE, MSE, and MAR are as follows:

(7) $$MAE_{class\text{\_}j}=\frac{1}{N}\sum_{i=1}^{N}\left|{{\hat{y}}_{i}}^{j}-{y_{i}}^{j}\right|$$

(8) $$MSE_{class\text{\_}j}=\frac{1}{N}\sum_{i=1}^{N}{\left({{\hat{y}}_{i}}^{j}-{y_{i}}^{j}\right)}^{2}$$

(9) $$MAR_{class\text{\_}j}=\frac{1}{N}\sum_{i=1}^{N}\frac{{{\hat{y}}_{i}}^{j}}{{y_{i}}^{j}}\times 100\%$$

where j∈[0,1] represents a category where 0 and 1 denote different classes. *N* is the total number of test samples, and ${{\hat{y}}_{i}}^{j}$ denotes the model’s classification result for class *j* in the *i*th picture. ${y_{i}}^{j}$ represents the ground truth annotation for class *j* in the *i*th image. The corresponding results are presented in Table 5.

The detection results in Table 4 confirm the effectiveness of the proposed improvements. Replacing the standard C2f module in the YOLOv8n backbone with the MSPA C2f module increased the precision by 0.8%. Incorporating the GD mechanism reduced the number of parameters by 41.8% and improved the inference speed by 9.3% while also producing a 0.8% gain in precision. The THead design achieved a precision of 87.0% without compromising the inference speed. When all three components are combined, MGDT-YOLO attains the highest detection accuracy, improving the precision by 2.6% and $mAP_{0.5}$ by 2.0% relative to those at the baseline; simultaneously, the model parameters, FLOPs, and model size are reduced by 58%, 46%, and 56%, respectively. Although a slight decrease in the inference speed was observed, MGDT-YOLO still satisfies the real-time detection requirements.

Considering that some methods’ accuracy gains are relatively modest (typically below 1%), we evaluated the robustness of these improvements further by repeating each configuration five times with the test set unchanged. Table 6 reports the mean ± standard deviation for the key metrics. The averaged precision values are as follows: baseline: 85.9±0.26%; MSPA C2f: 86.6±0.24%; GD: 86.8±0.27%; THead: 87.1±0.21%; M + GD: 87.0±0.23%; and M + GD + T: 88.4±0.21%. Similarly, the averaged $mAP_{0.5}$ values for the baseline and M + GD + T are 91.8±0.21% and 93.9±0.23%, respectively. Paired *t*-tests (baseline vs. each configuration; results provided in Table 6) show that the improvements in M + GD and M + GD + T are statistically significant (*p* < 0.05), confirming that the observed gains are reproducible and not attributable to random variation.

**Figure 18:**
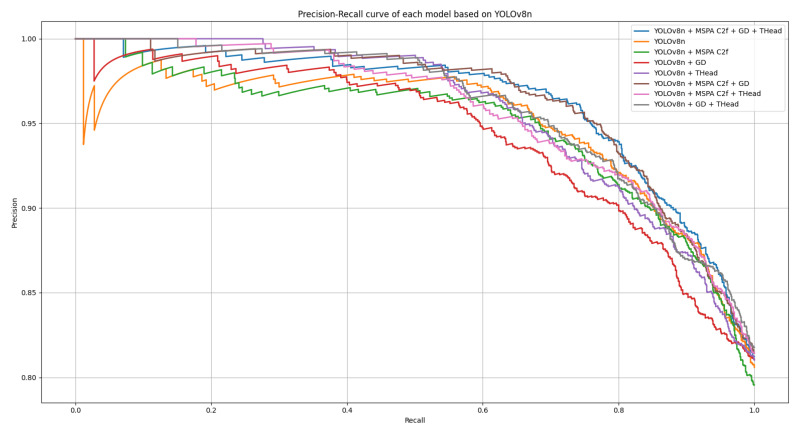
Precision–recall (P-R) curves.

For a more detailed evaluation of the model’s performance, precision–recall (P-R) curves are plotted for each model. As can be seen in Figure 18, MGDT-YOLO achieves a near-maximum area under the P-R curve while maintaining its lightweight design (the upper-right corner is the most critical).

In terms of the counting performance, a comparison and analysis of the data in Table 5 reveal that MGDT-YOLO accurately detects the most challenging class, class_0, which represents piglets that can move normally before weaning. Among the 841 samples in the test set, the model successfully detects 773 cases, missing 68. The average counting error is only 0.65 piglets, with a mean square error of 1.67, and the average detection accuracy is 91.91%, which is 2.26% higher than that of the baseline model. Counting such piglets is particularly difficult due to the variability in their numbers, possible multiple positions in the image, and potential occlusions. The model’s strong robustness for this class further demonstrates the effectiveness of the proposed improvements.

class_1, representing a piglet being born, exhibits relatively distinct characteristics and fixed positions and can only appear in quantities of either 0 or 1, making it easier to identify. This is reflected in the counting results, where the models achieve an average counting accuracy of over 95% for class_1. Among the 482 samples in the test set, most models miss fewer than 5 detections. This proves that the model can accurately detect sow delivery behavior, providing effective support for timely postpartum care for the sow and the protection of the newborn piglets.

### 4.3. Qualitative Results and Discussion

**Figure 19:**
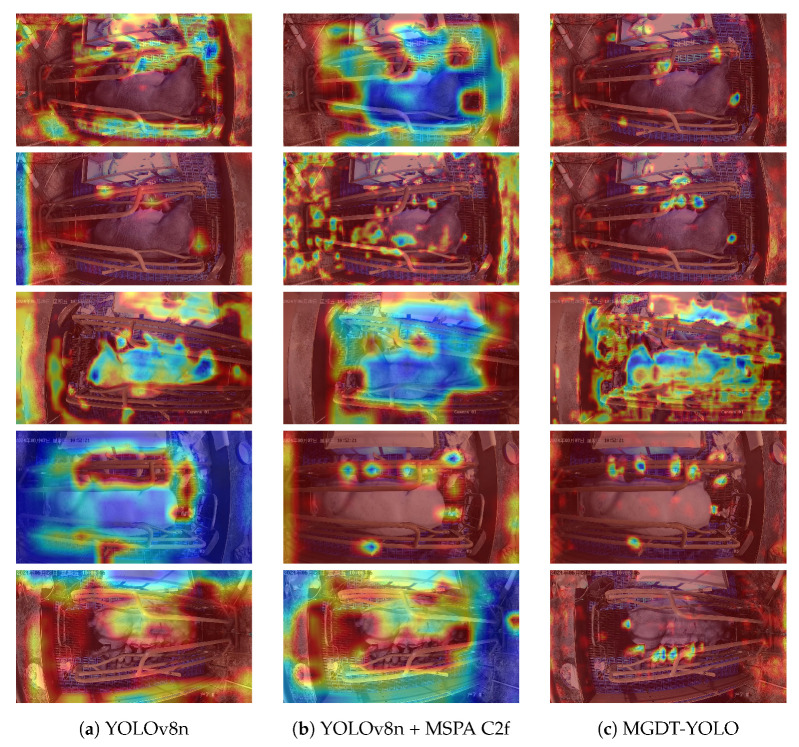
Visualization of feature activation maps for five representative test images using Grad-CAM++. The three columns correspond to (**a**) YOLOv8n (baseline), (**b**) YOLOv8n + MSPA C2f, and (**c**) MGDT-YOLO (proposed model). Each row represents a different test image. The heatmaps illustrate the regions of the image that contribute most strongly to the model’s detection results, highlighting the effect of the proposed improvements on the model’s focus and discriminative capability.

For a comprehensive evaluation of the proposed method, two complementary analyses were performed. Specifically, we first employed Grad-CAM++ [46] to visualize the feature activation maps of YOLOv8n before and after the proposed modifications were incorporated. All feature maps were extracted from the third-to-last layer of the network, which provided high-level semantic features and highlighted the regions most relevant to detection. As shown in Figure 19, integrating the MSPA C2f module directs the model’s attention more effectively to regions containing piglets while suppressing irrelevant background areas (blue regions). With all three improvements combined in MGDT-YOLO, the model demonstrates even greater sensitivity to target areas and achieves clearer separation of clustered piglets. This not only reduces the computational redundancy but also improves the detection accuracy.

**Figure 20:**
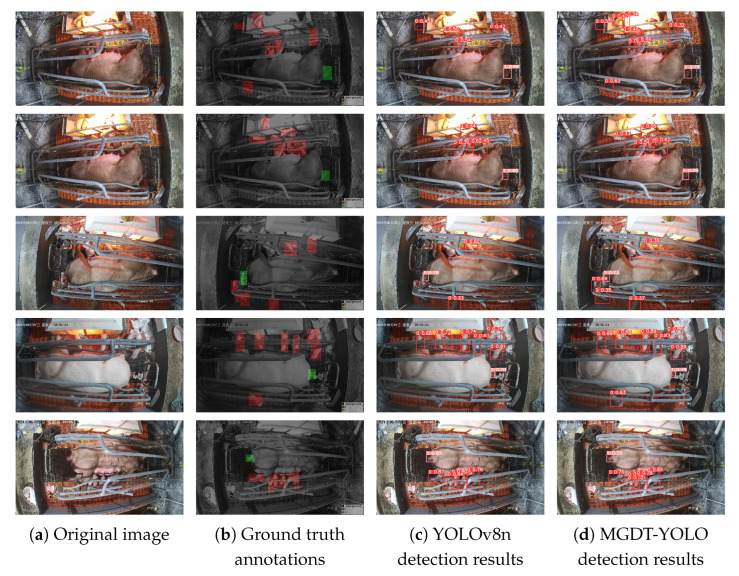
Comparison of the detection results for five representative test images. From left to right, the columns correspond to (**a**) the original images, (**b**) the ground truth annotations, (**c**) the YOLOv8n (baseline) detection results, and (**d**) the MGDT-YOLO (proposed model) detection results. Each row represents a different test image. The figure demonstrates how the proposed MGDT-YOLO improves the detection accuracy and reduces missed detections compared with those using the baseline model, particularly in challenging scenarios such as those with occlusion or overlapping piglets.

In addition, we compared the detection results under five representative challenging scenarios, as presented in Figure 20. By referring to the ground truth, YOLOv8n is observed to successfully detect piglets with distinctive features, such as those that are unobstructed or are currently being born from the sow’s birth canal (class_1). Nevertheless, some challenging regions still lead to missed detections—for instance, (a) the heavily occluded piglet in the lower-left corner and the overexposed piglet inside the heating box of the first image; (b) the piglet in the lower-left corner of the third image (occluded, lying posture); and (c) the piglet in the lower-left corner of the fourth image (occluded). By comparison, MGDT-YOLO effectively overcomes these issues, demonstrating a superior detection performance and confirming the validity of the proposed improvements. Despite these improvements, certain challenging cases remain. For example, in the lower-left region of the third image, this region contains one piglet from $class_{1}$ and three from $class_{0}$. The third piglet is almost entirely hidden by another piglet and the farrowing crate, with only its forelimb and forehead visible. Consequently, MGDT-YOLO fails to detect this piglet, resulting in a missed detection.

### 4.4. Deployment on Devices with Limited Computational Resources

Empirical studies have demonstrated that the direct deployment of deep learning models onto computational-resource-constrained devices is impractical. Benchmark tests reveal that the YOLOv8n architecture achieves merely 2.5 FPS (400 ms per image) on the Raspberry Pi 4B hardware [47]. To enhance the model’s real-time performance, we implemented model acceleration through Tencent’s open-source ncnn framework—a high-efficiency neural network inference engine specifically optimized for deploying deep learning models on mobile and embedded platforms. The workflow comprises three sequential phases: (1) the conversion of the trained PyTorch model (.pt) into TorchScript format; (2) the registration of custom operators (e.g., the TIM); and (3) generation of the architecture configuration (.param) and quantized weight (.bin) files. Notably, the converted MGDT-YOLO model demonstrates compact storage characteristics with a 35.2 KB param file and a 2.6 MB bin file (storing the quantized parameters). This processing pipeline enables the model to be directly loaded into C++, based on which we performed deployment tests on a resource-constrained device, specifically the Raspberry Pi 4B (4 GB of RAM).

**Figure 21:**
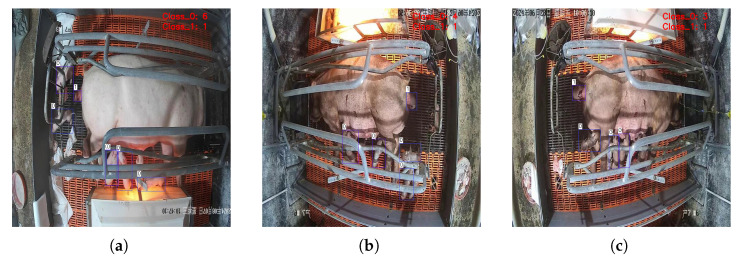
Visualization of the detection and counting results for MGDT-YOLO deployed on the Raspberry Pi 4B: (**a**) scene 1, (**b**) scene 2, (**c**) scene 3. The counting results are displayed in the top-right corner.

The experimental results show that the average inference time per image is 87 milliseconds, representing a greater than 4 times improvement compared to that in the pre-optimization stage. A more comprehensive performance evaluation is presented in Table 7. The model maintains a nearly equivalent accuracy to its original implementation, achieving 100% detection accuracy in monitoring sow farrowing events, with zero false positives for both types of piglets. The only two missed detections were later confirmed to be caused by severe occlusion. Visualization of the detection results is shown in Figure 21.

## 5. Conclusions

In this study, an object detection model based on YOLOv8n was proposed for piglet detection and counting in field farrowing crates. The model, named MGDT-YOLO, was designed to improve the accuracy and real-time performance of piglet detection and counting while reducing the computational load. To achieve this, the baseline network was enhanced according to the following three aspects: (1) A novel MSPA C2f module was proposed to strengthen the feature extraction capability of the network’s backbone. Compared with the original C2f module, MSPA C2f reduced the number of parameters and the computational complexity by over 86% while improving the detection accuracy by 0.8%. (2) An improved Gather-and-Distribute mechanism was introduced into the network neck to enhance the information flow efficiency between the local and global features. Compared with the baseline model, the improved model reduced the number of parameters and computational complexity by 23.46% and 41.85%, respectively. (3) The number of detection heads was reduced to one, and both the structure of the detection head and its sample assignment strategy were optimized. Compared with the baseline model, the optimized version achieved a 1.1% improvement in its detection accuracy while maintaining an efficient inference speed.

With the integration of the above three optimizations, the experimental results showed that MGDT-YOLO achieved only 41.55% of the parameter count and 53.09% of the computational complexity of the baseline model while reaching an average inference speed of 8.12 ms. Additionally, the detection accuracy and $mAP_{0.5}$ were improved by 2.6% and 2.0%, respectively, successfully fulfilling the goal of enhancing both the accuracy and real-time performance. Deployment tests on the Raspberry Pi 4B validated the effectiveness of the model’s lightweight design further, confirming its suitability for real-time applications to modern management in the pig industry.

Areas for further improvement remain in this study. First, while the proposed MGDT-YOLO improves the detection accuracy and reduces missed detections, its performance remains limited in cases of severe occlusion, such as piglets that are almost entirely hidden by other piglets or the farrowing crate. Partial occlusions are generally handled well, but extreme occlusions can still lead to missed detections, and thus, incorporating a GAN-based framework could improve the model’s reasoning performance further. Second, the dataset used in this study mainly includes images from a limited number of farrowing crates under controlled lighting conditions. Therefore, the model’s generalization to different farm environments, lighting conditions, or piglet breeds has not yet been fully validated. Third, due to the limited coverage of fixed-position cameras, deploying the model on mobile platforms could improve the system’s cost effectiveness and adaptability.

## Figures and Tables

**Table 1:** Comparison of computational complexity and parameters between MSPA C2f and C2f.

Model	Computational Complexity (MFLOPs)	Parameters (K)
C2f	2766.000	107.264
MSPA-C2f	375.196	14.402

**Table 2:** Training hyperparameter values of the proposed MGDT-YOLO.

Hyperparameter	Value
Training Epochs	100
Batch Size	16
Learning Rate	0.001
IoU Threshold	0.5
Data Augmentation	albumentations (flip, corp, hsv, brightness, mosaic, etc.)

**Table 3:** Comparative results of detection performance across different models.

Model	Precision (%)	Recall (%)	*mAP*_0.5_ (%)	mAP (%)	Params (M)	Model Size (MB)	FLOPs (G)	Inference Time (ms)
Faster R-CNN	39.26	95.53	74.50	48.50	99.252	404.1	188.416	65.4
SSD_Lite	63.12	86.48	69.60	48.30	3.403	15.3	2.754	8.0
TOOD	64.32	93.30	75.80	53.30	32.021	129.9	78.857	31.1
ATSS DyHead	68.57	94.28	79.10	60.60	38.892	160.7	43.559	46.1
YOLOX-tiny	86.25	49.28	72.10	36.60	5.033	59.6	3.199	8.4
Gold YOLO	83.40	92.00	91.62	64.20	5.620	49.6	12.1	2.29
YOLO11n	87.70	94.40	94.20	71.80	2.582	5.5	6.3	14.1
RT-DETRv2	-	-	94.80	75.30	40.444	141.0	132.7	15.2
Our work	88.50	91.70	93.80	70.30	1.249	2.7	4.3	8.0

**Table 4:** Comparative results of the proposed improvement methods. M stands for MSPA C2f, T stands for THead.

Method	MSPA C2f	GD	THead	Precision (%)	Recall (%)	*mAP*_0.5_ (%)	mAP (%)	Params (M)	Model Size (MB)	FLOPs (G)	Inference Time (ms)
Baseline	-	-	-	85.9	92.1	91.8	69.4	3.006	6.2	8.1	5.12
M	✓	-	-	86.7	90.6	92.1	69.0	2.532	5.3	6.8	8.22
GD	-	✓	-	86.7	86.6	89.6	59.5	1.748	3.5	6.2	4.64
T	-	-	✓	87.0	89.2	91.8	65.8	2.461	4.8	5.6	5.10
M + GD	✓	✓	-	86.9	92.4	93.9	70.9	1.341	2.9	5.7	7.91
M + T	✓	-	✓	87.0	88.9	91.0	62.2	1.987	3.9	4.3	8.01
GD + T	-	✓	✓	87.6	91.8	92.6	64.8	1.723	3.4	5.7	5.29
M + GD + T	✓	✓	✓	88.5	91.7	93.8	70.3	1.249	2.7	4.3	8.12

**Table 5:** The counting results of the proposed improvement methods. class_0 represents piglets capable of normal movement, and class_1 represents piglets in the process of being born from the sow’s birth canal.

Method	class_0						class_1					
	GT	TP	FN	MAE	MSE	MAR (%)	GT	TP	FN	MAE	MSE	MAR (%)
Baseline	841	754	87	0.68	1.85	89.65	482	479	3	0.02	0.03	99.37
M	841	751	90	0.67	1.81	89.29	482	478	4	0.04	0.05	99.17
GD	841	716	125	1.07	3.90	85.13	482	477	5	0.03	0.03	98.96
T	841	741	100	0.65	1.65	88.11	482	458	24	0.05	0.05	95.02
M + GD	841	770	71	0.63	1.63	91.55	482	480	2	0.02	0.03	99.58
M + T	841	719	122	0.63	1.52	85.49	482	459	23	0.05	0.05	95.23
GD + T	841	738	103	0.63	1.68	87.75	482	477	5	0.03	0.05	98.96
MGDT	841	773	68	0.65	1.67	91.91	482	478	4	0.02	0.03	99.17

**Table 6:** Ablation results reported as the mean ± std over five runs (test set unchanged). M: MSPA C2f; T: THead. Asterisks indicate p<0.05 vs. the baseline (paired *t*-test).

Method	MSPA C2f	GD	THead	Precision (%)	Recall (%)	*mAP*_0.5_ (%)	mAP (%)	Inference (ms)
Baseline	-	-	-	85.9±0.26	92.1±0.31	91.8±0.21	69.4±0.41	5.12±0.06
M	✓	-	-	86.6±0.24	90.7±0.33	92.2±0.26	69.1±0.46	8.23±0.08
GD	-	✓	-	86.8±0.27	86.7±0.42	89.5±0.31	59.6±0.61	4.63±0.05
T	-	-	✓	87.1±0.21	89.3±0.34	91.9±0.23	65.7±0.51	5.11±0.06
M + GD	✓	✓	-	87.0±0.23	92.3±0.32	94.0±0.24 *	70.8±0.36 *	7.90±0.09
M + T	✓	-	✓	87.0±0.25	88.8±0.37	91.1±0.31	62.3±0.54	8.02±0.09
GD + T	-	✓	✓	87.5±0.22	91.7±0.31	92.5±0.25	64.9±0.53	5.30±0.07
M + GD + T	✓	✓	✓	88.4±0.21 *	91.6±0.33	93.9±0.23 *	70.4±0.34 *	8.13±0.10

**Table 7:** Detection results of MGDT-YOLO deployed on the Raspberry Pi 4B.

Device	Ground Truth		True Positive		False Negative		False Positive	
	Piglet	Swine	Piglet	Swine	Piglet	Swine	Piglet	Swine
Raspberry Pi 4B	20	4	18	4	2	0	0	0

## Data Availability

The original contributions presented in this study are included in the article. Further inquiries can be directed to the corresponding author.
